# Deletion of *psbQ*’ gene in *Cyanidioschyzon merolae* reveals the function of extrinsic *PsbQ*’ in PSII

**DOI:** 10.1007/s11103-017-0685-6

**Published:** 2017-12-01

**Authors:** Maksymilian Zienkiewicz, Tomasz Krupnik, Anna Drożak, Wioleta Wasilewska, Anna Golke, Elżbieta Romanowska

**Affiliations:** Faculty of Biology, University of Warsaw, Miecznikowa 1, 02-096 Warsaw, Poland

**Keywords:** Stable red algae nuclear transformation, DTA toxin, *Cyanidioschyzon merolae*, Chloramphenicol acetyltransferase, PEG, PsbQ’, PSII mutants, PSII extrinsic protein deletion

## Abstract

**Key message:**

We have successfully produced single-cell colonies of *C. merolae* mutants, lacking the PsbQ’ subunit in its PSII complex by application of DTA-aided mutant selection. We have investigated the physiological changes in PSII function and structure and proposed a tentative explanation of the function of PsbQ’ subunit in the PSII complex.

**Abstract:**

We have improved the selectivity of the *Cyanidioschyzon merolae* nuclear transformation method by the introduction of diphtheria toxin genes into the transformation vector as an auxiliary selectable marker. The revised method allowed us to obtained single-cell colonies of *C. merolae*, lacking the gene of the PsbQ’ extrinsic protein. The efficiency of gene replacement was extraordinarily high, allowing for a complete deletion of the gene of interest, without undesirable illegitimate integration events. We have confirmed the absence of PsbQ’ protein at genetic and protein level. We have characterized the physiology of mutant cells and isolated PSII protein complex and concluded that PsbQ’ is involved in nuclear regulation of PSII activity, by influencing several parameters of PSII function. Among these: oxygen evolving activity, partial dissociation of PsbV, regulation of dimerization, downsizing of phycobilisomes rods and regulation of zeaxanthin abundance. The adaptation of cellular physiology appeared to favorite upregulation of PSII and concurrent downregulation of PSI, resulting in an imbalance of energy distribution, decrease of photosynthesis and inhibition of cell proliferation.

**Electronic supplementary material:**

The online version of this article (10.1007/s11103-017-0685-6) contains supplementary material, which is available to authorized users.

## Introduction

*Cyanidioschyzon merolae* is an extremophilic red microalga that dwells in moderately high temperatures (40–56 °C) and highly acidic (between pH 0.2–4) environments (Ciniglia et al. 2004). Being one of the most primitive algae, *C. merolae’s* cell possesses, among other organelles, a nucleus, one mitochondrion, and one chloroplast. Genomes of all three of these organelles were fully sequenced (Matsuzaki et al. 2004; Ohta et al. 1998, 2003).

Recently this simplicity has been utilized for genetic engineering of this alga, by the introduction of an exogenous antibiotic-resistance gene, effectively rendering it impervious to the toxicity of the respective antibiotic (Zienkiewicz et al. 2017a, b). Combination of three factors was crucial for achieving the stable genome or chloroplast mutant lines of *C. merolae*, capable of overexpressing an exogenous antibiotic resistance gene (Zienkiewicz et al. 2017a, b). These are as follows: application of endogenous promoters (ensuring high, continuous or variable gene expression), a codon usage optimization of the chloramphenicol resistance gene (*cat*) and an improvement of the previously used PEG-based DNA delivery technique. Additionally, a newly developed transformation procedure, utilizing biolistic bombardment could be used as an alternative. Nonetheless, these proceedings were insufficient for the undertaken attempts to generate a *psb*Q’ deletion lines. Most probably due to a number of illegitimate recombination events, constituting the predominant gene integration events (Zienkiewicz et al. 2017a). This observation largely agrees with the results of other researchers, showing that a proper double homologous recombination in nuclei of algae or higher plants is a very rare phenomenon indeed (Jasin and Berg 1988; Vergunst and Hooykaas 1999; Puchta 2002; Puchta and Hohn 1996; Paszkowski et al. 1988; Walker et al. 2005). Therefore, we decided to employ an upstream and downstream encoded genes of diphtheria toxin (fragment A—DTA), effectively flanking the entire length of the recombination sequence in the transformation vector, aiming at reduction of the number of illegitimate recombination events. The proper integration event, occurring by the means of specific double homologous recombination should result in rejection the *dta* gene sequences from both sides of the construct. Any illegitimate integration will allow either of the two *dta* genes to be expressed and in consequence lead to death of the transformed cell. Diphtheria toxin, a 58 kDa exotoxin secreted by *Corynebacterium diphtheriae*, can inactivate the ADP-ribosylation of elongation factor 2 (EF2), resulting in inhibition of protein synthesis and eventual cell death. The EF2 elongation factor is highly conserved among all kingdoms of life. The *C. merolae* eEF-2 (CMS428C) retains 78% identity with *Galdieria sulphuraria*, 67% identity with rice (*Oryza sativa*) or maize (*Zea mays*), and 62% identity with mouse (*Mus musculus*) or human (*Homo sapiens*), indicating that it may be inhibited by DTA in a similar way. Diphtheria toxin can be cleaved by trypsin into fragment A (193 aa) and fragment B (342 aa). Here, we have used the gene of the fragment A of diphtheria toxin (DTA), which is a 21 kDa protein, fully capable of inactivation of EF2, thereby inhibiting cellular protein synthesis (Pappenheimer 1977; Collier 1975). Previous studies have shown that the toxicity of the DTA protein is extremely high; a single molecule of DTA is able to cause cell death, but it isn’t toxic in the extracellular medium (Yamaizumi et al. 1978, Palmiter et al. 1987), therefore cross-toxicity is negligible. The applicability of the diphtheria toxin as a negative selectable marker, which promotes a specific site-directed recombination has been also widely investigated (Yuguang et al. 2006; Brockschnieder et al. 2004, 2006). For example, the transformation efficiency of embryonic stem cell (ES), generated by homologous recombination reached up to ~ 4%, whereas no such mutants have been identified without application of the DTA gene (Yanagawa et al. 1999). In other reports, the frequency of homologous recombinants varied significantly and was estimated at ~ 2% (Yagi et al. 1993), ~ 10% (McCarrick et al. 1993) or even ~ 50% (Yagi et al. 1990). Araki et al. (2006) showed that the negative selection with the DTA gene improves the transformation frequency by 2–4-fold for the Cre-Mediated Cassette Exchange in mice ES cells. McCarrick et al. (1993) used mice ES cells, neo gene and DTA toxin in transformation vector with one long 5′ flank (over 6 kb) and short 3′ (> 1 kb) flank and reported average ~ 10% of homologous recombinants. The first application of the DTA toxin in plant cells has been attempted for rice and allowed for an efficient gene targeting by homologous recombination (Tereda et al. 2002). The high similarity of the eEF-2 proteins and the fundamental character of the process by which the DTA toxicity arises (inhibition of protein synthesis) in *C. merolae* and rice, allow assuming that DTA is at least as toxic to *C. merolae* as it is to rice.

This method can be successfully applied for selective silencing or deleting of genes of interest i.e. the *psb*Q’ gene of an extrinsic subunit of PSII. The PsbQ’ protein is located in the vicinity of PSII oxygen-evolving complex (OEC) in red algae (Enami et al. 1998). It is a four-helix bundle, located underneath of the lumenal loop region of the CP43 protein in the vicinity of PsbV, yet on the opposite side of PsbO (Protein Data Bank coordinates 4YUU; Ago et al. 2016) in the PSII structure of *Cyanidium caldarium*. PsbQ’ does not interact directly with the OEC but is required for maximum efficiency of all extrinsic proteins assembly on PSII and for maximal activity of oxygen evolution (Enami et al. 1998), as assessed by an in vitro Release-Reconstitution experiment. Both *C. caldarium* and *C. merolae* are red extremophilic algae, relatively closely related, with *C. caldarium* having nearly twice the genome size of *C. merolae* (Matsuzaki et al. 2004; Toda et al. 1995), hence their protein structures can be used exchangeably as an approximation. The protein sequence of PsbQ’ from *C. merolae* is 68.8% identical with PsbQ’ from *C. caldarium*. Superimposed structure of *C. merolae* PsbQ’ modeled on the scaffold of *C. caldarium* PsbQ’ of revealed high structural homology between these two proteins (Supplementary Fig. S1). The average distances between the modeled structure residues and the scaffold residues are no greater than 0.5 Å (depicted in blue, Supplementary Fig. S1) with one residue, noticeably out of alignment, placed at the vertex of the loop between the second and the third helix (depicted in red, Supplementary Fig. S1). At this point the scaffold sequence appears to be discontinuous, however, the authors give no explanation as to why (Ago et al. 2016), nor to the partial only embedding of the PsbQ’ sequence within the observed electron density. PsbQ’ is located within ~ 30 Å distance from the OEC, what may exclude direct interactions between them (Supplementary Fig. S1), instead it probably functions more as a stabilizer of the OEC or assembly facilitator of the remaining extrinsic subunits (Enami et al. 1998). Its presence is not essential for PSII to maintain function, yet the lack of it diminishes PSII activity by ~ 50% and hinders PsbV and PsbU association with OEC. PsbQ’ gene has migrated to the nucleus by means of the yet unknown mechanism of gene transfer and it undergoes the nuclear mechanisms of regulation, perhaps constituting a mechanism of the nuclear control over the PSII assembly and activity, thereby control of photosynthesis.

Red algae are considered an intermediate evolutionary group of organisms between cyanobacteria and green algae or higher plants (Bricker and Ghanotakis 1996). The graduate appearance of derivative traits in the evolution of the photosynthesis can be perhaps best exemplified by the evolution of the sequence, structure, and composition of the extrinsic subunits of photosystem II. Cyanobacteria possess three extrinsic PSII subunits: PsbO (33 kDa), PsbV (23 kDa), and PsbU (17 kDa) of which PsbO is present in red algae along with three further subunits: PsbQ’ (20 kDa), PsbV (12 kDa), and PsbU (12 kDa) bearing only marginal resemblance to their cyanobacterial analogs (Ohta et al. 2003a, b). In green algae and higher plants, there are three extrinsic subunits: PsbO (33 kDa), PsbP (23 kDa) and PsbQ (17 kDa). Across all of these groups of organisms, only the PsbO subunit is the essential and the most conserved protein, which functions as the scaffold for OEC as well as for its cofactors and substrates (De Las Rivas et al. 2004). PsbO conserved function is held by some key elements of its structure and sequence (De Las Rivas and Heredia 1999), simultaneously the majority of its sequence may vary substantially and retain only 30% of total identity, as in the case of cyanobacteria and higher plants. The PsbQ’ subunit appears only in red algae as an additional fourth unit and was named after the higher plant’s PsbQ protein due to their similarity—in case of PsbQ’ (23.6 kDa) of *C. merolae* the most similar higher plant homologue is pineapple (*Ananas comosus*) PsbQ, retraining 30% sequence identity. In the course of evolution, both subunits of higher plant PsbQ and red algae PsbQ’ have been derived from cyanobacterial CyanoQ (Ifuku 2015). Similarly, the higher plant PsbP evolved from CyanoP. Red algae constitute an evolutionary mosaic, combining the ancestral PsbU and PsbV—lost in higher plants and derivative PsbQ’ without PsbP—perhaps lost after splitting of red and green algae lineages.

Here we report for the first time an improved method for highly selective and highly efficient production of *C. merolae* nuclear mutants by homologous recombination enhanced by DTA toxin. Additionally, we elucidate the role of the extrinsic PsbQ’ subunit of the PSII complex in the maintenance of photosynthetic activity and other physiological parameters by investigating Δ*psb*Q’ mutant of *C. merolae*.

## Results

### Transformation of *C. merolae*

**Fig. 1:**
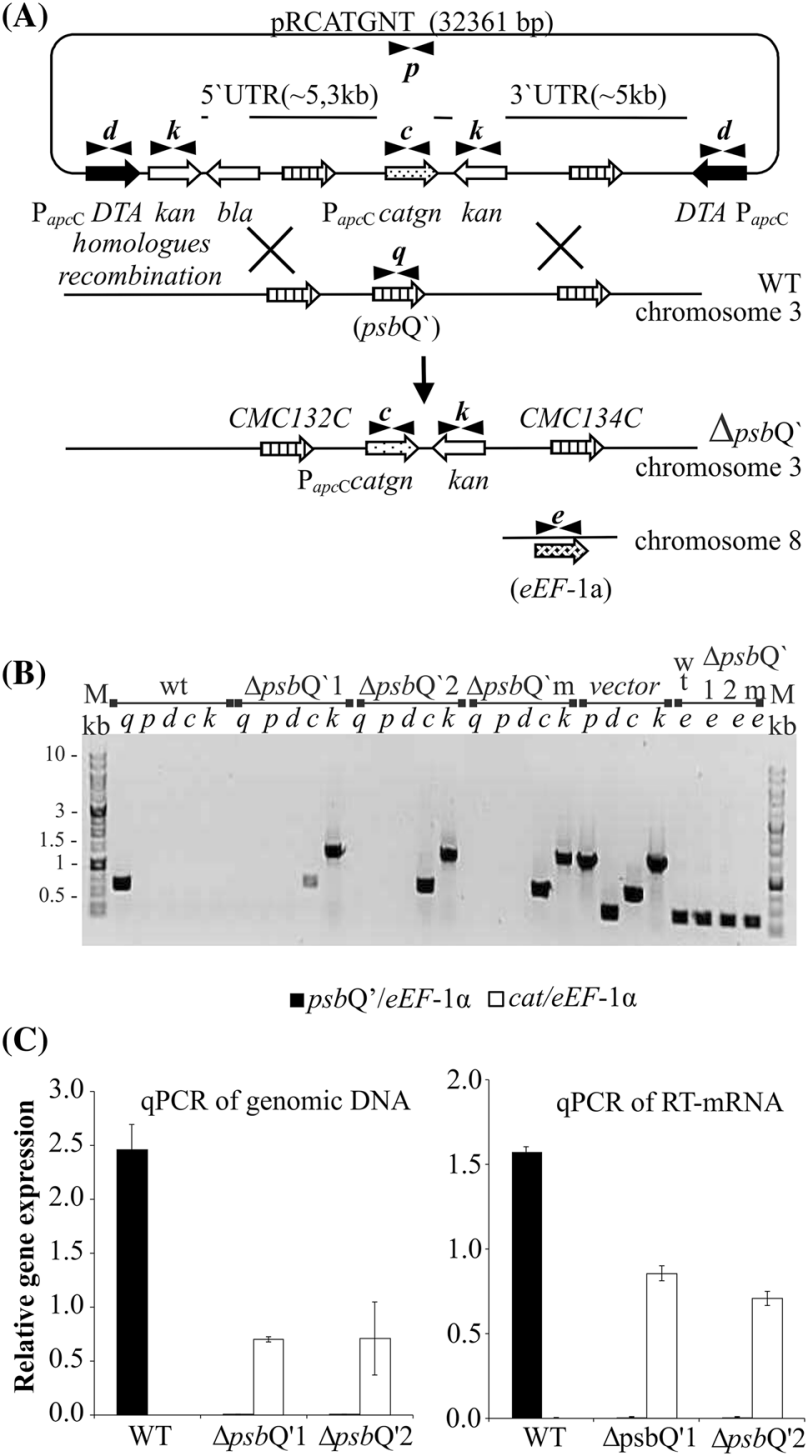
Scheme of *psb*Q’ replacement by *cat* gene and qPCR assessment of deleted or introduced gene in Δ*psb*Q’1 and Δ*psb*Q’2 mutants. The transformation vector (32,361 bp, **a**) was introduced to cells aiming a double homologues recombination, according to the scheme. The PCR experiment assessed the presence of selected markers (**a** and **b**): q, *psbQ*’; p, *ori*; d, DTA toxin; c, *cat* (chloramphenicol resistance gene); k, kanamycin resistance gene; e, *eEF-1a* control gene (**b**). The transformation vector was used as a control. The quantities of the *psb*Q’ (black bars) and *cat* (white bars) genes were expressed in ratios to the constitutive *ef1α* gene (**c**)

*Cyanidioschyzon merolae* is an organism capable of accepting and incorporating foreign DNA into its nuclear and chloroplast genome with an ease (Zienkiewicz et al. 2017a, b), resulting in possible unspecific integration event. To ensure higher specificity we decided to introduce a set of toxins, in addition to ~ 5 kbp long homologous flanks, located in the immediate neighborhood of the transfection cassette (Fig. 1). The full sequence of the transformation vector (pRCATGNT) was deposited in the GenBank under the accession number KY766997 (Supplementary Fig. S2). The complete vector, containing the *cat* gene (chloramphenicol resistance gene), the Δ*psb*Q’ mutation-carrying sequence, two homologous flanking regions (each ~ 5 kbp) and two toxin genes, was proliferated in *E. coli* host and isolated, pending *C. merolae* transfection. The osmotic shock mediated DNA delivery was performed as described before (Zienkiewicz et al. 2017a). The transformed cell-suspension was cultivated in normal growth condition for 3 days then the selective pressure was introduced. The selective conditions were retained for 2 months with regular exchange of the culture medium at 3 days interval and gradual increase of chloramphenicol pressure up to 600 µg mL^−1^. A sample of the highly resistant cells was spread on a Petri dish with solidified MA2 medium, supplemented with 200 µg mL^−1^ chloramphenicol and allowed to grow until single-cell colonies appeared. Further, two random colonies were moved to fresh liquid MA2 medium supplemented with 200 µg mL^−1^ of chloramphenicol and allowed to grow for 3 months with medium exchange. The precise information of the frequency of double homologous recombination was lost during the period of unselective growth, followed by a period of selective growth. Cells directly speeded on solid and selective plates did not exhibit any growth (Fujiwara et al. 2017). In this setup, the original proportions of chloramphenicol resistant/none-resistant cells were lost as the none-transformed cells died off in the selectable conditions. However, we report four independent attempts to performed this experiment with all of them yielding proper double homologous recombinants.

### Genetic and physiological characterization of *C. merolae* ΔpsbQ’ mutant cell lineages

**Fig. 2:**
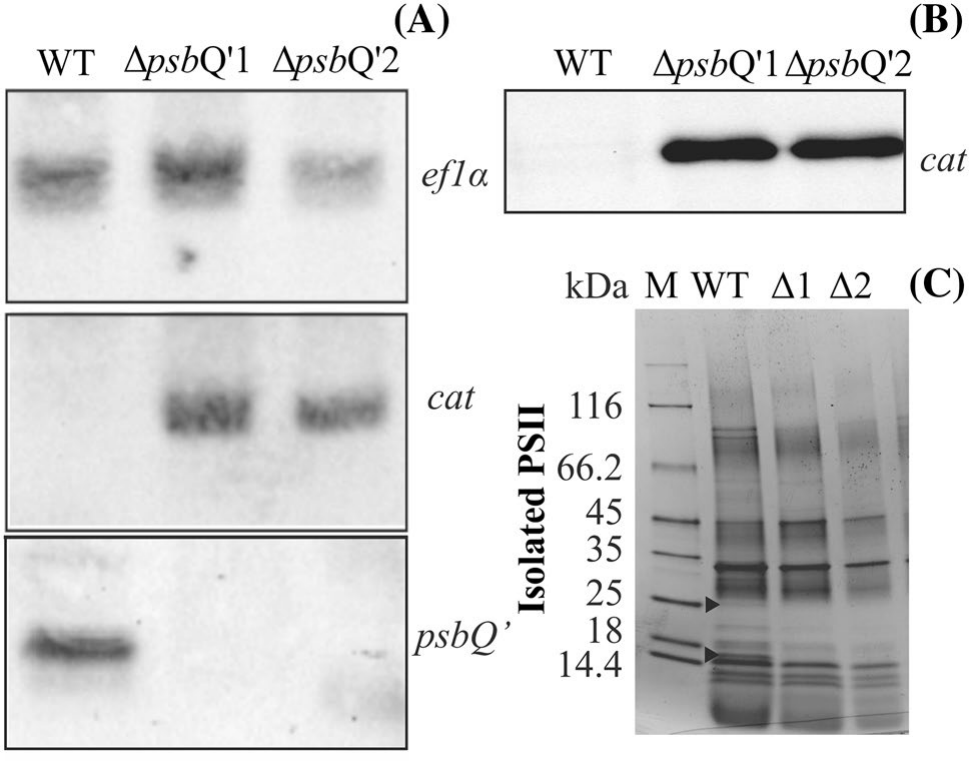
The genome and protein content analysis. For Southern blot analysis the total DNA was isolated from both mutants and the WT, digested with the HindIII (Thermo, USA) restriction enzyme, transferred onto a nitrocellulose membrane after agarose gel separation and finally hybridized with *psb*Q’ and *cat* gene-specific DIG probes (**a**). The constitutive *eflα* gene was used as a quality and quantity control. The western blot hybridization of the total cell lysate, separated on an SDS-PAGE gel and transferred onto an Immobilon-P membrane and treated with the anti-CAT antibody (**b**). Isolated PSII dimer samples (5 µg) were loaded on 12% SDS-PAGE gel and separated by protein electrophoresis (**c**). The Coomassie-developed gel shows two bands in the WT, vanishing in both mutants at ~ 23 kDa and ~ 17 kDa (marked with black triangles), roughly the size of PsbQ’ (23.614 kDa) and PsbV (16.607 kDa)

To confirm that the proper deletion of the *psb*Q’ gene has been achieved we have performed a series of exhaustive tests, aiming at ruling out any cells that might have acquired the resistance to chloramphenicol in an unspecific way. The PCR screening procedure (Fig. 1b) allowed us to confirm an incident of a proper double homologous recombination in both selected mutants (annotated as Δ*psb*Q’1 and Δ*psb*Q’2) and characterized them further. First, the isolated total DNA was tested for the presence of the *psb*Q’ gene, a fragment of the *ori* (a part of the transformation vector) sequence, the toxin sequence as well as the *cat* gene (chloramphenicol resistance gene) and the*kan* gene (kanamycin resistance gene) by standard PCR method (Fig. 1b). As expected, the *psb*Q’ gene amplification product was present only in the wild-type and was entirely absent in both selected mutant strains. The *cat* resistance gene was present in both mutants, ascertaining a high level of chloramphenicol tolerance. The additional screening factor—the diphtheria toxin wasn’t present in neither of mutants nor the wild type. The presence of kanamycin gene (a byproduct of cloning) suggests that one of the recombination events has occurred further in the flanking region, away from the *psb*Q’ locus (Fig. 1a). The same analysis *via* PCR performed on whole heterogeneous cell suspension, marked as ∆*psb*Q’m (mix) in Fig. 1b has revealed identical results, suggesting that the only cells, that did undergo the proper double homologous recombination event could survive the antibiotic pressure, what excludes any viability of illegitimate recombinants. Then, the exact contribution of the *psb*Q’ and *cat* genes in Δ*psb*Q’1 and Δ*psb*Q’2 was further analyzed in the total DNA and RT-mRNA (cDNA) by quantitative PCR (qPCR). The contribution of the *psb*Q’ gene in both mutants was negligible but in the WT it was ~ 2.5 and ~ 1.5 times higher than the reference gene *ef1α* in the total mRNA and DNA respectively (Fig. 1c). The *cat* gene and its transcripts contribution were both present at ~ 75% level of the reference gene in both mutants. A similar conclusion was drawn from DNA immunoblot (Southern Blot, Fig. 2a) experiment, where the total DNA of both mutants and WT was digested with HindIII enzyme, separated by agarose gel electrophoresis, reblotted on nitrocellulose membrane and hybridized with specific DIG probes, complementary to selected gene sequences of *psb*Q’, *cat* and *ef1α*—a constitutive gene, used as DNA quality and quantity control (Fig. 2a). The *psb*Q’ gene was detected only in the WT and *cat* only in mutant cells. The *ef1α* gene level was visibly, yet insignificantly lower in the Δ*psb*Q’2 mutant.

**Fig. 3:**
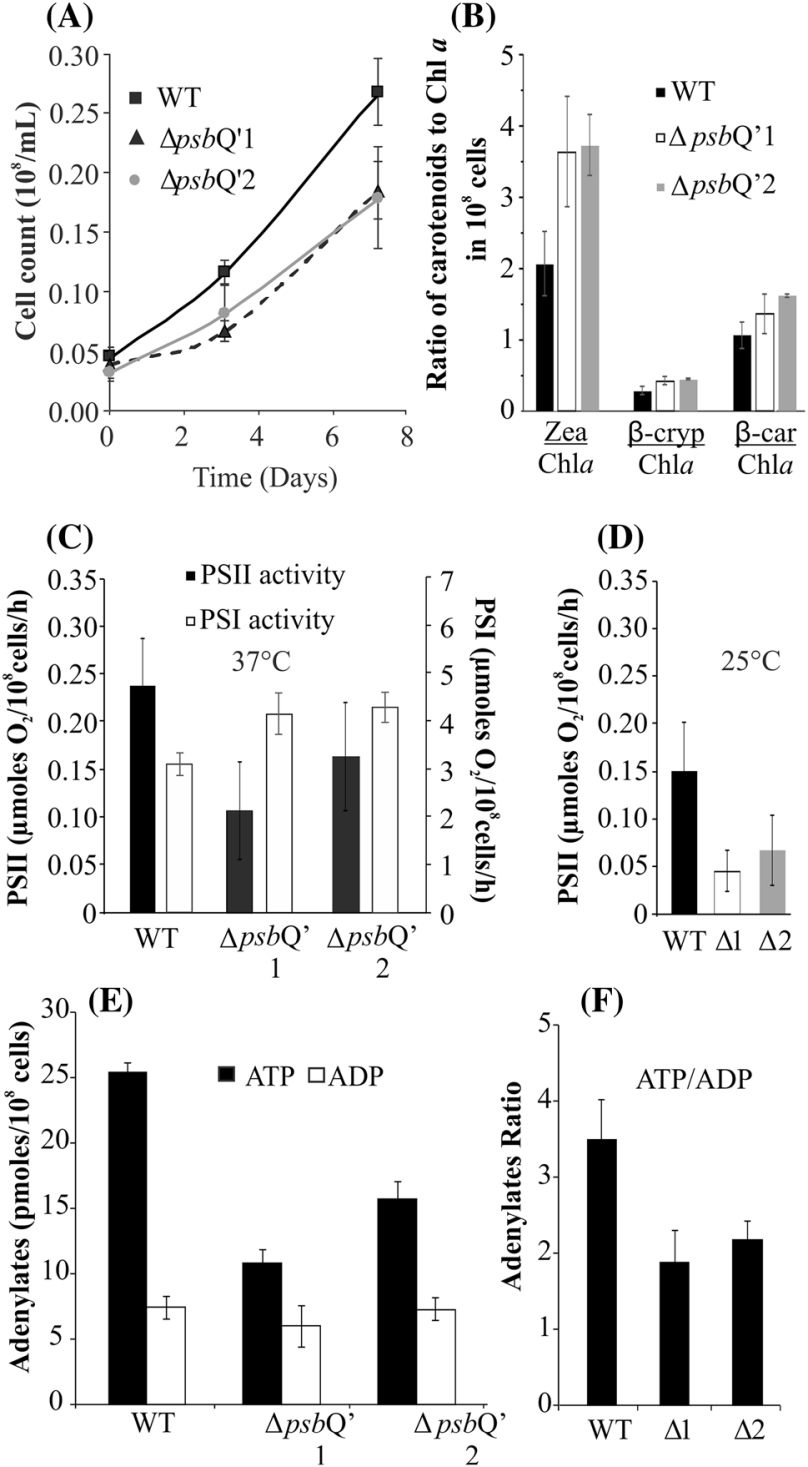
Cells physiology characteristics. Assessment of physiological changes, caused by deletion of the *psb*Q’ on physiological parameters. The growth dynamics was recorded as cell count (**a**) in cells without chloramphenicol. The carotenoid content from both mutants and the WT were separated on a C18 column by HPLC and expressed as the ratio of particular carotenoid to chlorophyll (**b**). The oxygen evolution by PSII and oxygen consumption by PSI was measured on an oxygen electrode illuminated with 500 µmoles photons m^−2^ s^−1^ at a temperature of 37 and 25 °C (**c, d**). The concentration of adenylates was measured in freshly harvested cells (**e**) and the energization (expressed as the ratio of ATP/ADP) of the mutant cells was at ~ 50–60% of the WT (**f**). All measurements were repeated three times

**Fig. 4:**
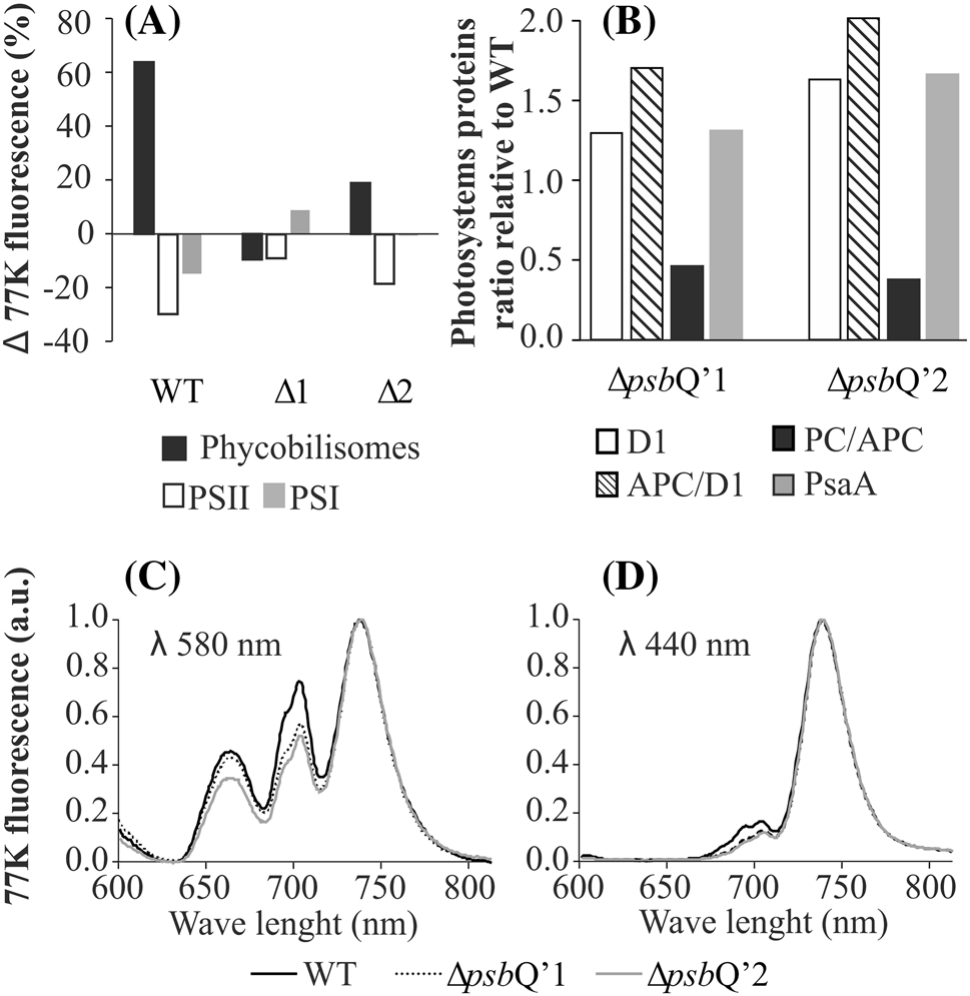
Cells fluorescence and antennae components. Mutant and the WT cells were exposed to 5 min pulse of 1000 µmoles photons m^−2^ s^−1^, than 77 K fluorescence spectra were recorded (before and after the pulse). Changes of relative fluorescence peak area were expressed as fraction of unexposed cells fluorescence (**a**). Western blot densitometric quantification of relative changes in protein abundance. Equal number of mutant and WT cells exhibits different proteins abundance, expressed as fraction of WT protein (**b**). 77 K fluorescence spectra were recorded for the phycobilisomes excitation wave λ = 580 (**c**), and chlorophyll *a* excitation wave λ = 440 (**d**)

To assess any phenotypical differences between WT and both mutants, samples of freshly grown cells were harvested and the protein composition was analyzed on an SDS-PAGE gel electrophoresis and the presence of the CAT resistance protein was confirmed by Western Blot (Fig. 2b). Both mutants exhibited slower growth than the WT and at the 7th day, both mutants were at ~ 65% of the WT cell count in the atmospheric CO_2_ concentration (Fig. 3a) and in the MA2 growth medium without chloramphenicol. Further, cell-extracted carotenoids were separated by the HPLC method (Fig. 3b), showing a significant increase (raised by twofold) in zeaxanthin production for both mutants with roughly unchanged levels β-cryptoxanthin as compared to the WT. Levels of β-carotene were increased by ~ 25% in both mutants. The oxygen evolving activities of both mutant lines and WT were measured on Clark-type oxygen electrode under 500 µmoles photon m^−2^ s^−1^ illumination at 37 and 25 °C. It was observed that both mutants: Δ*psb*Q’1 and Δ*psb*Q’2 exhibited lower levels of oxygen production in both temperatures, by ~ 50 and ~ 30% respectively, as compared to the WT (Fig. 3c, d). Similar, temperature-dependent activity of *C. merolae* was observed before (Nikolova et al. 2017). The mutant cells were more susceptible to temperature than the WT and a drop of the ambient temperature to 25 °C has diminished the activity of both mutants by ~ 50% while the WT retained as much as ~ 65% of its activity from 37 °C conditions. The activities of PSI were assessed by oxygen consumption on the Clark-type electrode in identical conditions as PSII, yielding increased activity of PSI in both mutants by ~ 25% (Fig. 3c). To assess the energization state of mutant cells the concentration of ATP and ADP was measured (Fig. 3e). The WT exhibited 3.5 times greater abundance of ATP than ADP, characteristic for a typical cell level. The abundance of ATP in both mutants was lower by 55 and 45% respectively and the ratio of ATP/ADP was ~ 50% of the wild-type (Fig. 3f). To establish the influence of PsbQ’ deletion on the antennae system the 77K fluorescence spectra were acquired. Cells of both mutants and the WT were collected into two sets of fluorescence cuvettes and one of these was irradiated with 1000 µmoles photon m^−2^ s^−1^ for 5 min. Samples were excited with 580 nm wavelength beam and spectra were collected in 600–800 nm range. The obtained spectra were deconvoluted and differences in areas underneath individual peaks were expressed as Δ of fluorescence intensity (Fig. 4a). It was observed that the WT showed over 65% increase of free phycobilisomes fluorescence after irradiation. This change was associated with over 30% decrease in PSII and 15% of PSI fluorescence. Both mutants exhibited much smaller changes in their fluorescence, with ΔpsbQ’2 showing only 20% rise in free phycobilisomes and 20% drop in PSII fluorescence. The ΔpsbQ’1 showed ~ 10% drop in phycobilisomes and PSII fluorescence, as well as ~ 10% increase in PSI signal (Fig. 4a). The changes in photosystems and antennae protein composition were approximated by semi-quantitative Western Blot method. An identical number of cells (10^6^) of both mutants and WT were separated on an SDS-PAGE, reblotted on a membrane and probed with antibodies against D1 (PSII core), PsaA (PSI core), APC (allophycocyanin) and PC (phycocyanin) (Supplementary Fig. S3). Protein-specific bands were quantified densitometrically (Supplementary Fig. S3) and expressed as the ratio of the mutant to the WT protein contribution (Fig. 4b). It was observed that PSI and PSII were more abundant by 25 and 50% in ΔpsbQ’1 and ΔpsbQ’2 mutants respectively, simultaneously, retaining identical ratio (PSI/PSII). The ratio of APC/D1 has increased by 75 and 100%, suggesting that about twice as many molecules of PSII were connected with APC on average in both mutants. The ratio of PC/APC was decreased by 50% in both mutants, indicating that the average length of phycocyanin rods was reduced by a factor of two in relation to the WT (Fig. 4b). The Western blot membranes were presented in Supplementary Fig. S3. The 77 K spectra of the WT and both mutants were normalized to the PSI (724 nm) fluorescence peak. The 580 nm excited samples showed elevated (by ~ 20%) level of PSII (680 nm) fluorescence in the WT (Fig. 4c). Similarly, the WT PSII fluorescence was also more intense when excited by 440 nm beam (Fig. 4d).

### Characterization of isolated PSII complex in ΔpsbQ’ mutants

**Fig. 5:**
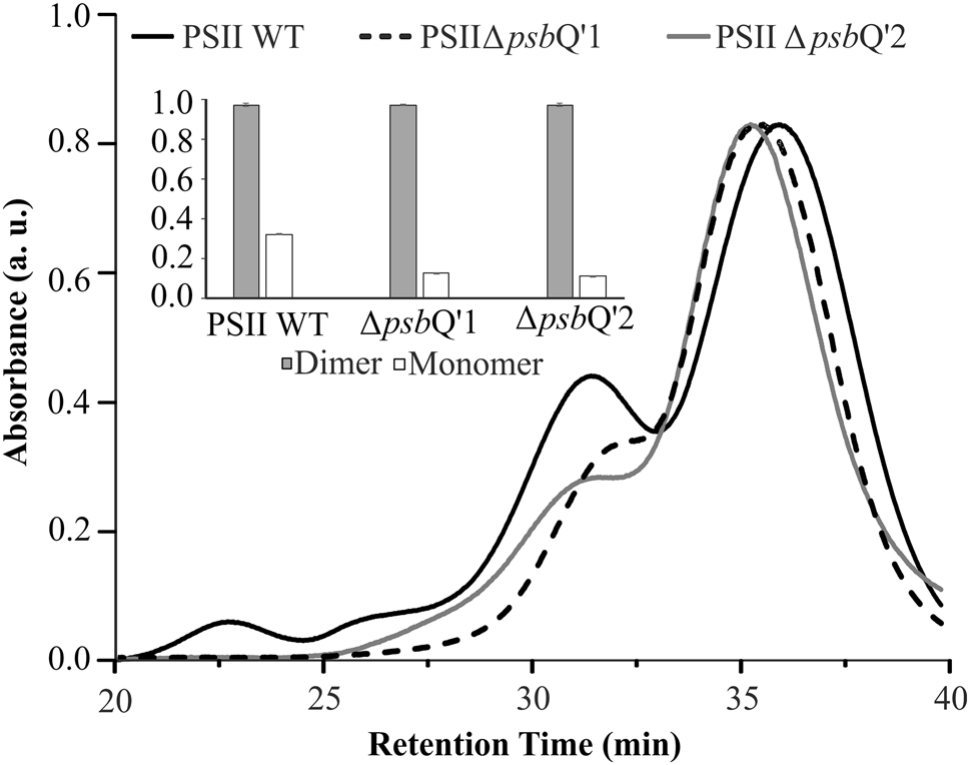
Contribution of monomers and dimers of *psb*Q’ PSII in deletion mutants. Pure PSII was obtained from solubilized thylakoids of WT and mutant cells, separated on an AEC column by HPLC. Monomers and dimers were separated in a linear gradient and eluted at retention time of ~ 33 min and 36 min, respectively. It was observed that either of the mutant PSII elutes ~ 2 min earlier than the WT. The relative proportions of monomer and dimer were assessed (inset). The WT thylakoids possess three times greater abundance of monomer than either of mutants

**Fig. 6:**
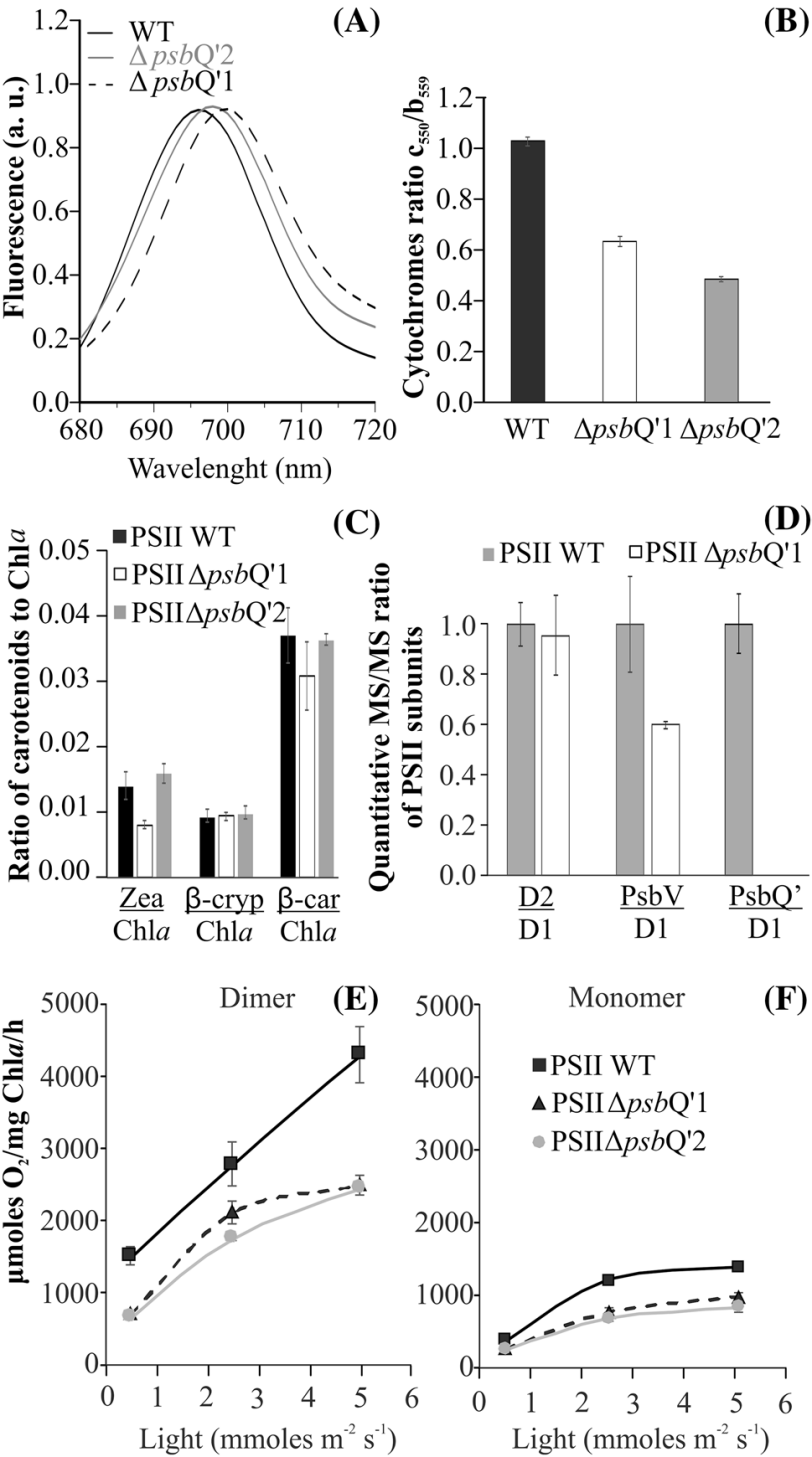
Characterization of isolated mutant PSII complexes. The 77 K fluorescence spectra were collected for WT and both mutants (**a**). It was observed that the λ_695_ peak of the WT PSII was red-shifted in both mutants up to λ_699_ in the Δ*psb*Q’1 and λ_697_ in the Δ*psb*Q’2. The contribution of cytochromes (*c*_550_ and *b*_559_) was established by redox difference spectroscopy (**b**). Carotenoids were extracted from 20 µg (Chl *a*) of the dimeric fraction of WT and both mutants PSII and separated on a C18 column by HPLC. The carotenoid content was expressed as the ratio of particular carotenoid to chlorophyll *a* (**c**). The relative contribution of PsbQ’ and PsbV extrinsic subunits was assessed by LC-MS/MS analysis of trypsin-digested whole PSII complex; analysis details are placed in Supplementary Table S3 (**d**). The oxygen evolving activity was measured on an oxygen electrode in white light illumination of 500–5000 µmoles photons m^−2^ s^−1^ for dimers (**e**) and monomers (**f**)

The PSII samples from WT and both mutants were isolated essentially as described before (Krupnik et al. 2013). The isolated thylakoids were solubilized in DDM (1% per 1 mg of chlorophyll) and separated on DEAE TOYOPEARL 650 M and 650 S resin-filled columns. Chromatograms were recorded and analyzed by the Clarity Chromatography Software (DataApex, Czech Republic). It was observed that both Δ*psb*Q’ mutant strains exhibited about three times lower contribution of monomeric PSII than the WT (Fig. 5). All chromatograms were deconvoluted by Microcal Origin 6.0 software and the relative contributions of monomer and dimer for all lineages were normalized (Fig. 5, inset). The pure PSII dimers (5 µg Chl *a*) from both mutants and the WT were loaded onto an SDS-PAGE, in order to identify protein bands of interest (Fig. 2c). It was observed that both mutants exhibited a lack of characteristic bands at the height of ~ 23 and ~ 17 kDa (present in the WT), corresponding to the PsbQ’ and PsbV with a molecular weight of 23.614 and 16.607 kDa, respectively. These bands were excised and analyzed by LC-MS/MS spectrometry techniques, allowing to confirm the presence of the PsbQ’ detected with the mascot score 368 and 41% coverage in MS/MS fragmentation peptides in the upper band. Similarly, PsbV was detected with mascot score 2703 and 76% coverage in MS/MS fragmentation peptides of the lower band. The measurement of 77 K PSII fluorescence showed a gradual shift of the characteristic 695 nm peak (Andrizhiyevskaya et al. 2005) towards the red end of the spectrum (Fig. 6a). The PSII Δ*psb*Q’1 mutant appeared to be shifted by nearly 5 nm, up to 699.5 nm while the PSII Δ*psb*Q’2 mutant peak migrated only by 2 nm. Then, the contribution of cytochrome *c*_550_ (PsbV) and *b*_559_ (PsbE) was examined by redox spectrometry. As it was established before (Krupnik et al. 2013) the stoichiometry of both cytochromes in the PSII complex was equimolar. It was concluded that the PSII Δ*psb*Q’1 mutant exhibited ~ 40% lower contribution of *c*_550_ in relation to *b*_559_ but the Δ*psb*Q’2 mutant cytochrome ratio appeared to be even lower, reaching ~ 50% of the WT contribution (Fig. 6b). Samples (10 µg of Chl *a*) of WT and both mutant PSII isolated protein were treated with acetone to extract carotenoids and chlorophyll. These pigments were when separated on the C18 column by the HPLC method and the content of particular carotenoids was analyzed (Fig. 6c). The contributions of particular carotenoids were expressed as the molar ratio of carotenoid to chlorophyll *a*. It was observed that the content of zeaxanthin in PSII Δ*psb*Q’1 was lower by ~ 50% in relation to the WT with simultaneously 10% lower content of β-carotene. The PSII Δ*psb*Q’2 protein was similar to the WT. The quantitative assessment of extrinsic subunits contribution was conducted for the Δ*psb*Q’1 mutant by LC-MS/MS, yielding ~ 40% lower abundance of the PsbV/D1 subunits ratio than in the WT (Supplementary Table S3). At the same time, the contribution of the PsbQ’/D1 was undetectable (Fig. 6d). The ratio of D2/D1 was measured for the WT and the mutant to confirm that the core units remained at the equimolar ratio. Due to different ionization properties of particular tryptic peptides, all ratios of the WT were normalized to unity and the mutant ratios were expressed as fractions of the WT ratios. The oxygen evolution activity was measured on a Clark-type oxygen electrode in 5 µg (Chl *a*) of PSII protein sample, under increasing (500–5000 µmoles photon/m^−2^ s^−1^) illumination. It was observed that the activities of both mutant-PSII dimers were lower by 45% in comparison with the WT (Fig. 6e) at the highest light intensity. Similarly, the isolated mutant-monomers were ~ 40% less active than the WT monomer (Fig. 6f).

## Discussion

The mutant cells of *C. merolae* were produced by PEG-mediated delivery of the transformation vector (pRCATGNT) and subsequent integration into the cellular genome via a double homologous recombination event (Fig. 1a). This method seemed to be highly efficient as we have had transformed four independent *C. merolae* cultures and in all cases, chloramphenicol-resistant cells had been obtained. A long period of selective growth might have caused an accumulation of cells, originated from a very small pool of initially transformed cells. The proper deletion of the *psb*Q’ gene sequence within cellular genomes was confirmed independently via Southern hybridization (Fig. 2a) as well as the Real Time PCR of the total DNA and the reverse-transcribed mRNA (Fig. 1c). The genetic analysis *via* PCR with a set of primers, designated to confirm the correct integration of the transformation plasmid with the genome, excluded the presence of the vector remnants (Fig. 1b) like the *ori* site or DTA (diphtheria toxin fragment A) genes as well as the target *psb*Q’ gene. Additionally, an SDS-PAGE and LC-MS/MS confirmed the absence of PsbQ’ protein within the ∆*psb*Q’1 and ∆*psb*Q’2 mutant (Fig. 2c). Interestingly, the same analysis, performed on whole heterogeneous cells has revealed identical results, suggesting that only the cells, which did undergo the proper double homologous recombination event were viable. Such efficiency, reaching indeed 100%, of *psb*Q’ sequence replacement with *catgn* sequence via double homologous recombination exceeds far beyond the efficiency obtained up to date. It was typically observed that the frequency of homologous recombination events increases together with the length of the homologous regions (Fujitani et al. 1995). Therefore, it can be expected that the frequency of double homologous recombination events could be increased by applying longer homologous flanks at both ends of the gene of interest in the transformation vector. Sufficiently long homologous regions could yield even up to 100% efficiency. Indeed, the length of both homologous flanks, targeting the *psb*Q’ locus in *C. merolae* genome was almost equal in size and had the length of ~ 5 and ~ 5.3 kb for 3′ and 5′ flanks, respectively. The entire length of the homologous sequence was over 10 kb and was terminated by two DTA toxin genes under the endogenous and efficient promoter of the *apcC* gene at both ends. Recently Fujiwara et al. (2017) reported high frequency (up to 70%) of homologous recombination in *C. merolae*. The authors attributed this to the length of homologous flanks (200 bp were required, 500 bp were sufficient). However, their heterogeneously grown transformants weren’t hindered by any deleterious effects of the introduced mutation. We anticipate, that most deletion mutations, introduced into *C. merolae*, may put it at some level of metabolic disadvantage and the proper mutants can be outcompeted by illegitimate mutants at the step of heterogenous growth. This propensity has been found to be the root problem of previous, unsuccessful attempts to select *psb*Q’ deletion mutants (Zienkiewicz et al. 2017a). The mechanism of DTA cellular toxicity involves inhibition of ADP-ribosylation of EF2 peptide elongation factor and high sequence similarity of EF2 protein across all kingdoms of life suggests that DTA is similarly toxic to all, plant or animal cells. The toxicity of DTA for tobacco plant cells was reported by Czako and An (1991) and confirmed additionally by Tereda at al. (2002) in rice. Up to date, there was no report calming a successful application of DTA toxin gene in algae. Therefore, we can hypothesize that the diphtheria toxin chain A is exceptionally toxic in small unicellular red algae like *C. merolae*. This toxicity allowed for highly specific selection of proper double homologous recombinants with the virtually total exclusion of illegitimately acquired resistance to chloramphenicol. Taking to account, that an identical (in respect to the length of the homologous regions) transformation vector, but without flanking DTA toxins, was unsuccessfully used before (Zienkiewicz et al. 2017a), we can confidently assign the improved selectivity to the introduction of DTA toxin genes. Further studies are planned in the future to establish, how reproducible and how universal might be the application of DTA toxin in combination with chloramphenicol resistance for enhancement of double homologous recombination events in *C. merolae* or other algae. The presented studies seem to indicate that this system can be very effective indeed, and in respect to small unicellular red algae, constituting an alternative to the CRISPR method.

The *psb*Q’ deletion strains of *C. merolae* were characterized by multiple and noticeable changes in cell physiology as well as the PSII complex function and structure. In *C. caldarium*, a closely-related red alga, a homologous tetra-helical structure of PsbQ’ protein (Supplementary Fig. S2) constitutes a ring of extrinsic proteins, ensuring the optimal function of OEC (Ago et al. 2016). High sequence similarity (68.8%) as well as the structural similarity of a modeled structure allowed to assume that the extrinsic subunits of *C. merolae* surround its OEC in similar if not identical fashion as in *C. caldarium*. Thus, the PsbQ’ interacts directly with PsbV (cytochrome *c*_550_) and PsbO, but there are probably no direct interactions between manganese-calcium cluster and PsbQ’. The Δ*psb*Q’ mutants were entirely depleted of PsbQ’ protein (Figs. 2c, 6d), what in turn caused lower binding efficiency of PsbV (cytochrome *c*_550_) subunit (Fig. 6b, e). These observations were suggestive of a cooperative binding mechanism of these extrinsic subunits, where all four subunits are needed for highest efficiency of binding (Kashino et al. 2006). Similar properties were observed before in *C. caldarium*, where the re-binding efficiency of dissociated subunits was the highest in the presence of all four of these subunits (Enami et al. 1998). The apparent lack of the PsbQ’ precipitated nearly 40% lower binding of PsbV causing a ΔPsbQ’/ΔPsbV and ΔPsbQ’ heterogeneity (Fig. 6b, e). Since the pure dimeric sample of PSII ΔPsbQ’ exhibited 40% lower activity (Fig. 6e) than the WT, it was expected that the ΔPsbQ’/ΔPsbV contributed mainly to the diminished activity, as PsbV interacts more closely with manganese-calcium cluster, hence the lack of PsbV exposes the manganese-calcium cluster and makes it more vulnerable to damage or dissociation. The introduced mutation caused a significant shift in the 77 K fluorescence from 695 to 699.2 nm. Since the 695 nm fluorescence arises from excitations that are irreversibly trapped on red-absorbing ‘690 nm’ chlorophylls of CP47 (PsbC) (Andrizhiyevskaya et al. 2005) and PsbQ’ interacts directly with CP43 probably without direct interaction with CP47, located on the opposite side of the PSII structure, the observed shift cannot be easily explained. We propose that the PsbQ’ might interact with CP47 from the other monomer of the dimer over the dimer’s interface. The precise mechanism of the shift origins is not known; however, it may be speculated that the structure of CP47 gains more degrees of conformational freedom upon deletion of *psb*Q’, what in turn disrupts the π-stacking of chlorophylls in its structure.

The introduced mutation had a very profound influence on the physiology of the cell. Perhaps most noticeably on the growth rate (Fig. 3a). As concluded before (Zienkiewicz et al. 2017b) the high level of chloramphenicol acetyltransferase (CAT, resistance protein) expression had no effect on the rate of *C. merolae* growth so the observed drop in cell proliferation should be associated with the inefficiency of PSII Δ*psb*Q’. We suggest, that the mutant cells have engaged several cellular mechanisms, leading it to upregulation of PSII activity. For instance, the cellular abundance of PSII was higher by 30–50% (Fig. 4b) and it was observed that the PSII Δ*psb*Q’ exhibited three times lower abundance of the monomeric form of PSII as compared to the WT (Fig. 5). Since the monomer exhibited only ~ 40% of the dimer activity, it can be proposed that the monomer/dimer ratio is a cellular mechanism of PSII activity regulation. Lower activity of PSII Δ*psb*Q’ might have caused a shift in monomer/dimer ratio, thereby rescuing the overall efficiency of PSII output. This regulation could occur via unrelated and unknown mechanism but it could also be facilitated directly by PsbQ’. The cross-interface interaction between PsbQ’ and CP47 could explain both: the shift in the 695 nm fluorescence of CP47 and diminished monomerization of the supercomplex. However, more data is needed to confirm such interactions. If so, the function of the PsbQ’ subunit could facilitate the nuclear control over PSII activity and hence the rate of photosynthesis. It was also observed that the level of zeaxanthin was reduced by 50% in the PSII Δ*psb*Q’1 (Fig. 6c). As stated before (Krupnik et al. 2013), zeaxanthin helps to dissipate the energy of the excited states of chlorophyll, thus the abundance of core zeaxanthin increases in the high-light stress conditions providing added protection. The reduction of the typical level of zeaxanthin could suggest upregulation of PSII complex activity. Lower abundance of the quencher (zeaxanthin) in PSII (Fig. 6c) might increase the efficiency of the transfer of the excited states into the PSII reaction center and improve the light-harvesting output in the applied growth conditions. Interestingly, the determination of the cellular level of carotenoids shows an opposite tendency to that of isolated PSII complex (Fig. 3b). The mutant cells have nearly doubled their level of zeaxanthin with 10–15% increase of β-carotene. This profile of cellular carotenoids changes could be typical for high-light stress condition (Krupnik et al. 2013). However, the light irradiation was moderate (50 µmoles photons m^−2^ s^−1^ ) and identical to the WT. Simultaneously, the assessment of oxygen evolution and consumption activity (Fig. 3c) showed 25% rise of activity PSI and a nearly 50% drop of PSII, respectively. Perhaps the apparent upregulation of PSI triggered a zeaxanthin-mediated dissipation of the excited states in PSI. On another hand, the cellular abundance of PSI protein in mutants has increased by 40–60%. It follows that the mutant cells have initiated a selective photoprotection mechanism, that is likely to dissipate the excited states of PSI but not PSII, yet the highly upregulated cellular activity of PSII Δ*psb*Q’ does not overcome the deleterious effects of *psb*Q’ deletion. Highly upregulated PSI is probably partially quenched by zeaxanthin but the consequently increased cyclic flow of electrons does not result in higher production of ATP (Fig. 3e). Typically, the chloroplast-produced ATP is momentarily consumed by the Calvin cycle for production of phosphotrioses and in effect cellular storage materials (starch). In consequence, it does not constitute a significant fraction of cellular ATP. The energization level, expressed as the ratio of ATP/ADP is derivated from the mitochondrial oxidative respiration and further, availability of storage materials, limited for the Δ*psb*Q’ mutants nearly twice. Perhaps some of the most apparent physiological adaptations of the mutant cells were changes in the antennae system. *C. merolae* possesses phycobilisomal antennae associated with PSII and likely with PSI, additionally PSI is connected with 4 membrane-bound light harvesting systems (Lhcr). Shortly, phycobilisomes are built of phycocyanin (a protein harboring phycobilin chromophores) rods, connected to allophycocyanin core, further still, connected to PSII. Mutants exhibit nearly twice the amount of APC/D1 ratio (Fig. 4b), suggesting that two times as many PSII particles are connected to phycobilisomes. However, the ratio of PC/APC in mutants is just a half of that in WT, showing perhaps shorter PC rods in mutant PSII. Phycobilisomes are known to detach from the PSII moiety upon a pulse of light. The WT phycobilisomes disconnected easily from PSII, after an exposure to a light pulse, releasing large quantities of free phycobilisomes (Fig. 4a). Simultaneously, the fluorescence intensity of PSII dropped by 30%, likely caused by the loss of efficient antennae system. Only a small changes were observed in PSI, suggesting that little if any phycobilisomes had detached from PSI. Functional association of phycobilisomes with PSI is yet to be demonstrated. The Δ*psb*Q’ mutants show much less flexibility in rearranging their antennae. The likely reason is that mutant phycobilisomes are shorter and unable to collect light as efficiently as the WT (Fig. 4c), hence the light-induced detachment is abolished.

We propose that the diminished activity of PsbQ’/PsbV-depleted PSII is unable to deliver sufficient amount of reductants for the Calvin cycle. This might result in reduction of storage materials production and slower proliferation of cells. Likely, cells compensate for the mutation with upregulation of PSII activity by mechanisms of increased dimerization and core-protein cellular abundance as well as assembling shorter phycobilisomes (less likely to detach) and depletion of zeaxanthin-related quenching of the excited states in PSII. Simultaneously, the increased abundance of PSI results in higher activity, which might be quenched by the higher cellular level of zeaxanthin. Thus, the increased cyclic electron flow might result in higher chloroplast production of ATP but it isn’t efficiently used for CO_2_ fixation and production of storage materials or is used up locally, for others chloroplast energetic needs. The inefficiency in production of starch and other storage materials might cause a decrease in the level of cellular/mitochondrial ATP. The Δ*psb*Q’ mutant cells highly upregulate PSII and downregulate PSI, disrupting the typical flow of the harvested light energy and in consequence the balance of photosynthetic substrates and products.

## Conclusions

Overall, we report a highly efficient method for transformation of *C. merolae*. The application of DTA toxins under efficient P_*apc*C_ promoters allowed to highly increase the previously reported selectivity (Zienkiewicz et al. 2017a), that relied only on long homologous flanks and CAT selection. Our improved method may constitute an alternative to the CRISPR method in the transformation of *C. merolae*.

## Experimental procedures

### Cell cultures

*Cyanidioschyzon merolae*, 10D (NIES-1332, Unialgal, Clonal and Non-axenic) strain was obtained from Microbial Culture Collection (mcc.nies.go.jp, Tsukuba, Japan) and was used throughout this study. Cells were grown in MA2 liquid medium (Minoda et al. 2004) in a glass vessel under continuous white light (50 μmoles photons m^−2^ s^−1^ ) at 42 °C or on Petri dishes filled with MA2 medium, solidified by addition of 0.4% gellan gum (Phytagel™, Sigma, Germany) (Minoda et al. 2004) or 0.75% agar (Basica LE, Prona, EU).

*Escherichia coli*, strain DH5α (genotype: F-Φ80*lac*ZΔM15 Δ(*lac*ZYA-argF) U169 *rec*A1 *end*A1 *hsd*R17 (rK-, mK+) *pho*A *sup*E44 λ-thi-1 *gyr*A96 *rel*A1) were used for construction of transformation vectors (Hanahan et al. 1983). Bacterial cells were cultured in liquid LB medium (1% Bacto tryptone, 0.5% yeast extract, 1% NaCl at 37 °C) or on Petri dishes with LB medium solidified by addition 1% agar. For a selection of transformed cells, the medium was supplemented with an appropriate antibiotic (100 µg mL^−1^ of ampicillin, 25 µg mL^−1^ of tetracycline or 30 µg mL^−1^ of kanamycin).

### Plasmid construction

The construction of transformation vector pRCATGNT (32,361 bp, GenBank KY766997) involved generation of several interim plasmids and their detailed description was given in the Supplementary Material in Table S1 and Supplementary Fig. S1. The pRCATGNT is a low-copy-number plasmid with IncFII replicon, derived from p1658/97 (Zienkiewicz et al. 2007, 2013, Gene Bank Acc No. AF550679) and contains 5’UTR and 3’UTR of *C. merolae* psbQ’ gene separated by *cat*gn cassette under promoter of the *C. meroalae* gene *apc*C. The 5’UTR-PapcCATGN-*kan*-3’UTR fragment, re-cloned from pCCATGN kan (Zienkiewicz et al. 2017a), has been flanked by two *Diphtheria* toxin genes (Freeman 1951) under *apc*C promoter sequences.

### Nucleic acid isolation from *C. merolae* cells

DNA was isolated from *C. merolae* using standard CTAB in situ Hybridization procedure described earlier (Schwarzacher and Heslop-Harrison 2000) and RNA isolation was performed by conventional methods (Fujiwara et al. 2009).

### List of primers used in this study

The sequence of primers used in this study was presented in Supporting tables: Supplementary Table S2.

### Enzymatic manipulations of DNA

All enzymatic manipulations on DNA, such as restriction digestion, blunting of cohesive termini using T4 Polymerase or the Klenow fragment or ligation were performed according to protocols supplied by the manufacturers.

### PCR

PCRs were performed according to manufacturer’s protocols, supplied with the Phire Plant Direct PCR kit containing, Plant Phire Hot Start II DNA Polymerase (Thermo Fisher Scientific Inc., Waltham, USA) or DreamTaq DNA Polymerase, (Thermo Fisher Scientific Inc. USA).

### qPCR study

The real-time quantitative PCR assays were carried out on the PikoReal 96 Real-Time PCR System (Thermo Scientific, USA). Primers were designed in the Primer Quest program (http://eu.idtdna.com/PrimerQuest/Home/Index). Each reaction was carried out in the reaction mixture containing: 1× concentrated Real-Time 2×HS-PCR Master Mix SYBR A (A&A Biotechnology), forward and reverse specific primers (100 nM each), the DNA template [in three selected concentrations, decreasing five or four times (e.g. 15, 3 and 0.6 ng per well), each in a duplicate] and water in final volume of 10 µL. Reactions were performed with an initial denaturation step of 95 °C for 3 min, followed by 45–50 cycles of denaturation (at 95 °C for 15 s.) and primer annealing-extension (at 60 °C for 30 s.) steps. Fluorescence was acquired during the annealing-extension step of each cycle. Subsequently, the melting point temperature analysis was performed in the range of 60–95 °C. The quality of results was evaluated based on the expected C_t_ differences between the three DNA concentrations as well as the product melting curves. Rare outlying results were omitted in these calculations. Three concentrations of DNA allowed to calculate individual efficiencies for each primers pair and normalize all the results to one DNA concentration, common to all genes. The amount of each target gene was calculated by a modified ΔC_t_ method, with a geometric mean of two common genes C_t_s (Vandesompele et al. 2002).

### Southern blot analysis

The transfer of the HindIII-digested DNA from the agarose gel to a nitrocellulose membrane was performed by a conventional alkaline method using 20× SSC buffer (3 M NaCl, 0.3 M sodium citrate, pH 7.0). DIG-labeled DNA fragments were prepared by PCR using DIG Probe Synthesis Kit (Roche) with specific primers (catgnF and catgnR hybridizing with the *catgn* sequence, ef1F and ef1R hybridizing with the *ef1*-a sequence, psbQF and psbQR hybridizing with the *psb*Q’ sequence) and used as hybridization probes. The DIG was detected with alkaline phosphatase (AP)-conjugated anti-DIG antibody (Roche) and CDP-Star (Roche). The signals were visualized with the luminescent image analyzer ChemiDoc XRS + System (Bio-Rad, USA) or X-ray film (Kodak BioMax XAR Film, Sigma-Aldrich, Germany) was exposed to the membranes for 24–48 h and developed with the Cerastream Kodak developer solution (Sigma-Aldrich, Germany).

### SDS-PAGE and immunoblotting analysis

Mutant and control cells or PSII protein samples were harvested from fresh cultures, grown in MA2 medium without chloramphenicol by spinning down 1 mL of cell cultures for 5 min at 2000×*g* and resuspending in the resuspension buffer (20 mM Hepes-NaOH pH 7.6, 5 mM EDTA and 330 mM sucrose). Chlorophyll concentration was quantified spectrometrically (UV-1800 Shimadzu, Japan) by absorption measurement at λ_663_ of chlorophyll extracted with 80% (v/v) acetone. Numerical values were derived from the Beer–Lambert equation, extinction coefficient ε = 86.3 (L g^−1^ cm^−1^) and the dilution factor (×200). Alternatively, cells were counted with the Neubauer chamber as an average of at least ten counts. Cells and chloroplast samples were solubilized in denaturing buffer [0.25 M Tris–HCl (pH 6.8), 0.4% (w/v) SDS, 10 M urea, 2% (v/v) 2-mercaptoethanol and 20% (v/v) glycerol] and mixed together in 1:1 (v/v) ratio. Proteins were separated on 15% gels by Laemmli-type SDS-PAGE method (Laemmli 1970). Gel wells were loaded with 0.2–1 μg of Chl *a* or with an equivalent of 10^6^ cells and run under constant voltage (75 V). Following electrophoresis, polypeptides were electro-transferred on a PVDF-membrane (Towbin et al. 1979) and probed with rabbit anti-CAT (specific to chloramphenicol acetyltransferase) antibodies (Sigma, Germany) or with anti-APC (dil. 1:1500), anti-PC (dil. 1:1700), anti-D1 (dil. 1:10,000) and anti-PsaA (dil. 1:1000) rabbit antibodies (Agrisera, Sweden), specific to the selected components of the antennae systems. Bands were visualized binding of secondary, goat anti-rabbit antibody, conjugated with HRP (dil. 1:25,000) and by enhanced chemiluminescence method with the SuperSignal^®^ West Pico Stable Reagent (Thermo, USA) according to standard procedures using ChemiDoc System (Bio-Rad, USA).

### Coomassie blue staining of polyacrylamide gels

After electrophoresis gels were incubated for 2 h or O/N in buffer I (50% ethanol, 2% H_3_PO_4_) and washed with mili-Q water 3 × 20 min. Next, gels were incubated for 1 h in buffer II (34% methanol, 17% (NH_4_)_2_SO_4_, 2% H_3_PO_4_) and after that, ~ 1–2 mg of Coomassie powder (Coomassie Briliant Blue G-250, Bioshop Canada Inc., Canada) was added. Incubation was continued until protein bands appeared.

### Determination of ATP content in whole cells

The cell samples were harvested from fresh cell cultures grown to OD_680_ = 0.2 in MA2 medium without chloramphenicol. The cells were centrifuged at 16,9000*g* in 4 °C for 5 min in the dark and immediately ground in liquid nitrogen. The powder obtained was treated with 10% (v/v) HClO_4_ and left for 5 min on ice. The ice-cooled samples were centrifuged at 10.000 g for 2 min and aliquots of the supernatants were brought to pH 7.0 by adding 1 M triethanolamine in 5 M KOH. After 30 min on ice, the precipitated KClO_4_ was pelleted (10,000*g* for 2 min), and the adenylate contents were measured in the supernatants. ATP was determined by the firefly luciferase method (Gardeström and Wigge 1988). ADP was converted to ATP by pyruvate kinase (Boehringer, Mannheim, Germany) and determined as above. Each measurement was calibrated with an addition of ATP standard. The measurements were repeated at least three times in three to four separate experiments.

### LC-MS/MS identification of PSII proteins

PSII complexes (17 µg) were precipitated with cold (− 20 °C) acetone (1:4, v/v) and dissolved in 0.1% (w/v) RapiGest reagent (Waters, USA) in50 mM ammonium bicarbonate. Following reduction and subsequent alkylation of cysteine residues with10 mM DTT and50 mM iodoacetamide, proteins were digested with MS grade trypsin (Sigma-Aldrich, Germany) for 12 h at 30 °C. Reaction was terminated by the addition of trifluoroacetic acid to 1% (v/v) final concentration and resulting samples were centrifuged (13,000*g*, 10 min), filtered with Costar Spin-X filter (0.22 µm) and then supplemented with bovine serum albumin (BSA, Sigma, Germany) tryptic digest (922 fmoles, Waters, USA) as an internal standard. Peptides were analyzed by nano-UPLC-tandem mass spectrometry employing Acquity nano-UPLC coupled with a Synapt G2 HDMS Q-TOF mass spectrometer (Waters, USA) fitted with a nanospray source and working in MS^E mode under default parameters as described previously (Drożak et al. 2013, 2015). Briefly, products of PSII protein digestion (1.5 µg) containing BSA tryptic peptides (83 fmoles) were loaded onto a Waters Symmetry C18 trapping column (20 mm × 180 µm) coupled to the Waters BEH130 C18 UPLC column (250 mm × 75 µm). The peptides were eluted from columns in a 1–85% gradient of acetonitrile in water (both containing 0.1% formic acid) at a flow rate of 0.3 µL min^−1^. The peptides were directly eluted into the mass spectrometer. Each sample was chromatographed and analyzed three times. Data were acquired and processed using MassLynx version 4.1 software (Waters, USA) and ProteinLynx Global Server version 2.4 software (Waters, USA) with a false discovery rate of 4%, respectively. To identify and quantify proteins, the complete *C. merolae* proteome was downloaded from NCBI protein database, manually supplemented with BSA amino acid sequence (P02769), randomized, and used as a data bank of the MS/MS software.

### Carotenoid composition analysis

Analytical high-pressure liquid chromatography (HPLC), using the Shimadzu Prominence System with PDA detector, (Shimadzu, Japan) was performed according to a modified method of Krupnik et al. (2013) using a maximum flow rate of 1 mL min^−1^. and a Bionacom 3000 C18 column (Bionacom, UK). Pigments were extracted from cells (harvested from fresh cultures at OD = 0.2 without chloramphenicol) and PSII samples (0.5 mg Chl) with a 1 mL ethanol. The volume of cell suspension or PSII protein solute was no greater than 1/4 of the extraction mixture. Cellular and protein debris was removed by 10 min. centrifugation at 4 °C. The extract was concentrated in a SpeedVac at 30 °C centrifuge until it dried out. Samples (20 µg Chl) were dissolved in 50 µL of acetonitrile: triethylamine (99.9:0.1 v/v) and loaded onto the C18 column that was previously equilibrated with phase A (acetonitrile: methanol, 6:2 v/v). Pigments were separated with a linear gradient of 0–75% of phase B (ethyl acetate), starting at the 10th min of the run. The content of each carotenoid species was expressed as a molar ratio of a specific carotenoid and chlorophyll *a*. Concentration was calculated as the area under the pigment-corresponding peak. The molar attenuation coefficients at the wavelength λ = 436 nm in acetonitrile were as follows: 91.7 mM^−1^ cm^−1^ for Chl *a*, 133.3 mM^−1^ cm^−1^ for zeaxanthin, 131 mM^−1^ cm^−1^ for β-cryptoxanthin, 134.6 mM^−1^ cm^−1^ for β-carotene and 28.5 mM^−1^ cm^−1^ for pheophytin (Davies 1976). Rare incidences of chlorophyll degradation resulted in the appearance of pheophytin, which molar contribution was added to the moiety of chlorophyll.

### Purification of PSII

Purification of the dimeric PSII complex was performed according to a modified protocol of Adachi et al. (2009), using HPLC system (Sykam Chromatography, Germany) with 50 mL preparative injection loop. Thylakoids (1 mg mL^−1^ Chl) were solubilized with 1% (w/v) DDM (Roth, Germany) by gentle agitation at 4 °C for 40 min. in the dark. Solubilized membranes were centrifuged at 104,200×*g* for 30 min at 4 °C in ultracentrifuge (Beckman, USA) and the syringe-filtered supernatant was applied onto a DEAE TOYOPEARL 650 M column equilibrated with buffer A (40 mM MES-KOH pH 6.1, 3 mM CaCl_2_, 25% (w/v) glycerol) supplemented with 0.03% DDM. Loaded column was washed with 2 column volumes of buffer A and proteins were eluted with buffer B [500 mM NaCl, 40 mM MES-KOH pH 6.1, 3 mM CaCl_2_, 25% (w/v) glycerol]. Crude PSI and PSII were eluted with three step-gradient (1st step: 2 CV and 0% B, 2nd step: 3 CV and 18% B, 3rd step: 3 CV and 46% B). The obtained fraction of PSII was desalinated on preparative desalting column, filled with Sephadex G25 resin and buffer A, as the carrier buffer. Crude PSII was loaded onto a DEAE TOYOPEARL 650 S column equilibrated with buffer A. Pure PSII was eluted in step gradient (1st step: 2 CV and 0% B, 2nd step: 2 CV and 5% B) and followed by linear gradient 5–15% of buffer B to separate PSII monomers and dimers. PSII dimer fraction was concentrated using VivaSpin-20 (100K MWCO) concentrators (Sartorius Stedim, Germany) to at least 2 mg mL^−1^ Chl *a*, and stored upon snap-freezing at − 70 °C until further use.

### PSII and PSI activity measurement

Functional activities of PSII dimers (5 μg Chl) were measured using an oxygen Clark-type electrode (Hansatech, UK). Measurements were performed at 25 or 37 °C in buffer A (40 mM MES-KOH pH 6.1, 3 mM CaCl_2_, 25% (w/v) glycerol) in the presence of 0.125 µM 2,6-dichloro-*p*-benzoquinone (Sigma, Germany) and 125 µM potassium ferricyanide (POCH, Poland) as the exogenous electron acceptors. For measurement of cellular PSII activity, a cell suspension at OD_680_ = 0.2 from a fresh culture without chloramphenicol was used with 0.5 mM Na_2_CO_3_ (Chempur, Poland) as a source of CO_2_. Samples (1 µg mL^−1^ Chl) were illuminated with the standard white light intensity of 500–5000 µmoles photons m^−2^ s^−1^, for PSII protein or cells with 500 µmoles photons m^−2^ s^−1^, using a KL 2500 LCD white light source (Schott, Germany). Activities were calculated from the initial rates of oxygen evolution curve.

Cellular activities of PSI were measured using an oxygen Clark-type electrode (Hansatech, UK). Cell cultures at OD_680_ = 0.2 and 1 mL volume were spun down at 5000 rpm in 30 °C and resuspended in buffer D (40 mM Tris–HCl pH 8.0, 3 mM CaCl_2_). Cells were incubated in the electrode chamber and in the dark for 10 min at 37 °C in the presence of 2 µM methyl viologen (MV, 1,1′-dimethyl-4,4′-bipyridinium dichloride, Sigma, Germany), 2 µM DCPIP (2,6-Dichlorophenolindophenol, Koch-Light Laboratories, UK) 5 µM DCMU (3-(3,4-dichlorophenyl)-1,1-dimethylurea, Sigma, Germany) and 0.5% Triton^®^ X-100 (Acros Organics, USA). Subsequently, white light (500 μmoles photons m^−2^ s^−1^) was turned on and 60 µM ascorbate (Roth, Germany) was added. Activities were calculated from the initial rates of oxygen consumption curve. The background activity was measured likewise in the absence of cells. Cells counts were measured with the Neubauer chamber as an average of at least ten counts.

### 77 K fluorescence, RT absorbance, and redox spectroscopy

The low temperature (77.5 K) fluorescence spectra were acquired using Allegiant Technologies Cary Eclipse Fluorescence Spectrophotometer with a cryostat (Oxford Optistat DN2, UK). About 5 µg Chl *a* of pure PSII or cells was dissolved in 1 mL of buffer A, placed in a fluorescence cuvette and frozen in liquid nitrogen. Frozen cuvette was placed in the cryostat sample-holder and within the optic path of the fluorimeter. The sample was left to reach the precise temperature of 77.5 K and spectra were acquired within the 600–800 nm fluorescence range with 440 nm excitation beam. All RT absorbance measurements were carried out using Shimadzu UV 1800 spectrometer. The content of cytochrome *c*_550_ and cytochrome *b*_559_ was established by preparation of 5 mL of 0.01 mg mL^−1^ (Chl) PSII solution. Approximately 1 mL was taken to baseline the spectrometer in the range between 600–500 nm. Subsequently, a grain of ferrycyanide (K_3_[Fe(CN)_6_], POCH, Poland) was added, mixed and left for 30 s before acquiring a spectrum of a fully oxidized pool of cytochromes. A fresh sample was taken for full reduction with a grain of sodium dithionite (Na_2_S_2_O_4_, POCH, Poland). The sample was mixed and incubated for 30 s before spectrum was collected. To ensure complete oxidation or reduction of cytochromes pool an additional grain of ferrycyanide or dithionite was added before reacquiring spectra. The differential spectrum was calculated by subtracting the oxidized spectrum from the reduced spectrum. Cytochromes contribution was calculated from absorption values at specific wavelengths for cytochrome *c*_550_ and cytochrome *b*_559_ and known extinction coefficients (Kaminskaya et al. 2005) 27 and 25.1 mM^−1^ cm^−1^ respectively. Each measurement was repeated three times.

## Electronic supplementary material

Below is the link to the electronic supplementary material.

Supplementary material 1 (DOCX 20 KB)

Supplementary material 2 (DOCX 18 KB)

Supplementary material 3 (DOCX 18 KB)

Supplementary material 4 (DOCX 635 KB)

Supplementary material 5 (DOCX 109 KB)

Supplementary material 6 (DOCX 1052 KB)
