# Supplementary material for: Deletion of psbQ’ gene in Cyanidioschyzon merolae reveals the function of extrinsic PsbQ’ in PSII
Source: Plant Mol Biol. 2017 Dec 1;96(1):135–49. doi: 10.1007/s11103-017-0685-6 (PMC5778172; doi:10.1007/s11103-017-0685-6)
Supplement: Supplementary file 1 — Supplementary material 1 (DOCX 20 KB) [file 11103_2017_685_MOESM1_ESM.docx]

**Table S1. Plasmids constructed and used in this study.**

| **Plasmid name** | **Plasmid size**  **(in bp)** | **Remarks/plasmid construction** |
| --- | --- | --- |
| pACYC184 | 4245 | A commercially available vector. Here, used as a source of *tet and cat*  gene sequences. |
| pJET1.2 | 2974 | A commercially available vector (Thermo Fisher Scientific). Here used for cloning DNA fragments with blunt ends. |
| pKSBS P*_mc1_* DTA | 4987 | A derivative of pBluescript II KS (Stratagene) with DTA toxin gene under *mc1* promoter (Araki et al. 2006). The plasmid was used as a source of DTA toxin sequence. The plasmid is a gift from professor Kimi Araki. |
| pET28a(+) | 5369 | Commercially available vector. Here used as a source of *kan* gene sequence. |
| pR | 13654 | The p1658/97 derivative (Acc No. AF550679. Zienkiewicz et al. 2007) with incFII replicon of p1656/97 ensuring low copy number of plasmid molecules in *E. coli.* Detailed construction of the pR plasmid described in Zienkiewicz et at. (2013). |
| pCCATGN | 13770 | Derivatives of pACYC184 with 5`UTR and 3`UTR of psbQ` gene of *C. merolae* (coordinates in the chromosome 3 310641-316006 bp for 5`UTR and 316707-321727 bp for 3`UTR) and *catgn* gene under the control of *apc*C gene promoter. Detailed construction of this plasmid (pCCATGN. but without *kan* cassette. described in Zienkiewicz et al. (2017a). |
| pCCATGNkan | 15161 | The pCCATGN with *kan* cassette introduced into EcoRV restriction site of the plasmid. The *kan* sequence (1391 bp) was amplified in PCR with the use of kanF kanR primers and pET28a(+) as a matrix. An intermediate in procedure of creation of final *C. merolae* transformation vector. |
| pBSKS P*_apc_*_C_ DTA | 4943 | The pKSBS P*_mc1_* DTA derivative, in which *mc1* promoter sequence of *DTA* gene was replaced by promoter sequence of the *apc*C gene of *C. merolae*. The 615 bp PCR DNA generated by apcNcoI apcSalI primers and *C. merolae* genome as matrix was introduced into pKSBS P*_apc_*_C_ DTA by SalI and NcoI restriction enzymes. An intermediate in procedure of creation of final *C. merolae* transformation vector. |
| pJET1.2 P*_apc_*_C_DTA | 4943 | pJET 1.2 with integrated 1971 bp PCR fragment with P*_apc_*_C_DTA module generated with apcPsI and DTASacII primers and plasmid pKSBS P*_apc_*_C_ DTA as a matrix. |
| pJET1.2 P*_apc_*_C_DTA kan | 6336 | pJET1.2 P*_apc_*_C_DTA but with the *kan* cassette introduced into the SmaI site of the plasmid. The *kan* cassette (1391 bp) was amplified in PCR by kanF kanR primers and pET28(+) as a matrix. An intermediate in procedure of creation of final *C. merolae* transformation vector. |
| pBSKS P*_apc_*_C_ DTAx2 kan | 7726 | pKSBS P*_apc_*_C_ DTA derivative in which second P*_apc_*_C_ DTA fragment was introduced in opposite direction to the first *DTA* gene copy. The 3362 bp PCR fragment with P*_apc_*_C_ DTA – *kan* cassette, generated by apcPsiI and DTASacIIs primers and pJET1.2 P*_apc_*_C_DTA *kan* as matrix was digested with SacII and PsiI and introduced into the double digested (with SacII and PsiI) plasmid pKSBS P*_apc_*_C_ DTA. |
| pRDTA | 16667 | The pR derivative with introduced P*_apc_*_C_-*DTA*-*kan*-P*_apc_*_C_-*DTA* module amplified in PCR with the use of apcPsiI and apcSalI primers and plasmid pKSBS P*_apc_*_C_-DTA x2 kan as a matrix. The pR plasmid was digested with PsiI and SalI and ligated with digested with PsiI and SalI PCR DNA fragment (5339 bp). |
| pRCATGNT  GenBank  Accession no:  KY766997 | 32361 | Final plasmid used for transformation of *C. merolae*. Derivatives of pRDTA in which 5`UTR PapcCATGN–*kan*-3`UTR of psbQ` module (obtained with the use of NotI digestion of the pCCCATGN kan) was integrated into NotI restriction site of the plasmids pRDTA. Additionally the *tet* cassette of pACYC184 was introduced into SwaI restriction site of the plasmid. The *tet* cassette (1366 bp) amplified in PCR with the use tetF and tetR primers and pACYC184 as matrix. |
