# Supplementary material for: Deletion of psbQ’ gene in Cyanidioschyzon merolae reveals the function of extrinsic PsbQ’ in PSII
Source: Plant Mol Biol. 2017 Dec 1;96(1):135–49. doi: 10.1007/s11103-017-0685-6 (PMC5778172; doi:10.1007/s11103-017-0685-6)
Supplement: Supplementary file 2 — Supplementary material 2 (DOCX 18 KB) [file 11103_2017_685_MOESM2_ESM.docx]

**Table S2. Primers used in this study.**

| **Primer name** | **5`3` sequence** | **Remarks** |
| --- | --- | --- |
| apcCNcoI  apcCSalI | CTGA**CCATGG**TCAACGAACGAAGAAAC  CTCT**GTCGAC**CGACGAGAACGTATAAGGA | For plasmid construction - the *apc*C promoter sequence of *C. merolae.* In bold - sites recognized by NcoI and SalI restriction enzymes respectively. |
| apcPsiI DTASacIIs  DTASacII | ATAT**TTATAA**CGACGAGAACGTATAAGGAGTGC  TCGTA**CCGCGG**GGATCAGGATCCCCC  TCGTA**CCGCGG**GGATCAGGATCCCCCGGGCT | For plasmid construction - P*_apc_*_C_ DTA modules in plasmids (see Figure S1 and Table S1). In bold - sites recognized by PsiI and SacII restriction enzymes respectively. |
| tetF  tetR | GGTACCCAAATGTAGCACCTGAAGTC  CCCGGGAATTCTTGGAGTGGTGAATC | For plasmid construction - the *tet* cassette. |
| kanF  kanR | CCAACCCGGTAAGACACGAC  GCCGATTTCGGCCTATTGG | For plasmid construction - the *kan* cassette For testing of the presence *kan*  gene copy in *C. merolae* mutant lines. |
| psbQF  psbQR | TCCAACTGCAACCAAAGG  ATGCCAACAGTGGACATC | For testing of the presence psbQ` gene in *C. merolae* mutant lines. |
| pRoriF  pRoriR | GAGGTGCATATCTGTCTGTC  GGCCCACTTAAACCGTATAG | For testing of the presence plasmid pRCATGNT remnant (origin of plasmid replication) in *C. merolae* mutant lines. |
| catgnF  catgnR | GGTACCATGGAGAAGAAGATTACGGGTTACA  AGGCCTTTACGCACCGCCCTGCCAT | For testing of the presence *CATGN* gene in *C. merolae* mutant lines. |
| dtaF  dtaR | GGTCAAAGTGACGTATCC  CACACCACAGAAGTAAGG | For testing of the presence *DTA* toxin genes in *C. merolae* mutant lines. |
| ef1F  ef1R | GGCTTTGTATGCGGAGACAG  CCGCCTCTTTCTTGTTGACC | For testing of the presence *eEF-1a* gene in *C. merolae* – internal control of the amounts of the DNA given as a matrix in PCR. |
