# Supplementary material for: Deletion of psbQ’ gene in Cyanidioschyzon merolae reveals the function of extrinsic PsbQ’ in PSII
Source: Plant Mol Biol. 2017 Dec 1;96(1):135–49. doi: 10.1007/s11103-017-0685-6 (PMC5778172; doi:10.1007/s11103-017-0685-6)
Supplement: Supplementary file 3 — Supplementary material 3 (DOCX 18 KB) [file 11103_2017_685_MOESM3_ESM.docx]

**Table S3. Quantification of the extrinsic subunits by LS-MS/MS.**

|  | **Protein** | **PLGS Score** | **coverage (%)** | **Amount (fmol)** | **Prot/D1 ratio** | **Prot/**$\overline{\mathrm{Prot}_{\mathrm{WT}}}$ | **Prot fraction** | **Err** |
| --- | --- | --- | --- | --- | --- | --- | --- | --- |
| WT | PsbQ’ | 54664 | 55.2 | 1682.6 | 1.20 | 0.948 | 1 | 0.118 |
|  |  | 43265 | 52.9 | 990.1 | 1.44 | 1.135 |  |  |
|  |  | 65058 | 52.9 | 2203.0 | 1.16 | 0.916 |  |  |
|  | PsbV | 69263 | 65.3 | 6141.8 | 4.39 | 1.085 | 1 | 0.188 |
|  |  | 90857 | 65.3 | 3144.3 | 4.57 | 1.130 |  |  |
|  |  | 100768 | 65.3 | 6009.8 | 3.17 | 0.783 |  |  |
|  | D2 | 10896 | 25.5 | 1398.9 | 1.68 | 0.988 | 1 | 0.085 |
|  |  | 11176 | 21.3 | 687.2 | 1.85 | 1.090 |  |  |
|  |  | 12076 | 23.3 | 1895.0 | 1.56 | 0.920 |  |  |
| Δ*psb*Q’1 | PsbV | 98477 | 65.3 | 5552.8 | 2.38 | 0.589 | 0.598 | 0.013 |
|  |  | 131347 | 76 | 6112.8 | 2.45 | 0.607 |  |  |
|  | D2 | 12596 | 25.5 | 2328.6 | 1.59 | 0.933 | 0.955 | 0.157 |
|  |  | 14598 | 25.5 | 2485.1 | 1.37 | 0.809 |  |  |
|  |  | 21327 | 25.8 | 1454.6 | 1.9 | 1.122 |  |  |
