# Supplementary material for: Deletion of psbQ’ gene in Cyanidioschyzon merolae reveals the function of extrinsic PsbQ’ in PSII
Source: Plant Mol Biol. 2017 Dec 1;96(1):135–49. doi: 10.1007/s11103-017-0685-6 (PMC5778172; doi:10.1007/s11103-017-0685-6)
Supplement: Supplementary file 4 — Supplementary material 4 (DOCX 635 KB) [file 11103_2017_685_MOESM4_ESM.docx]

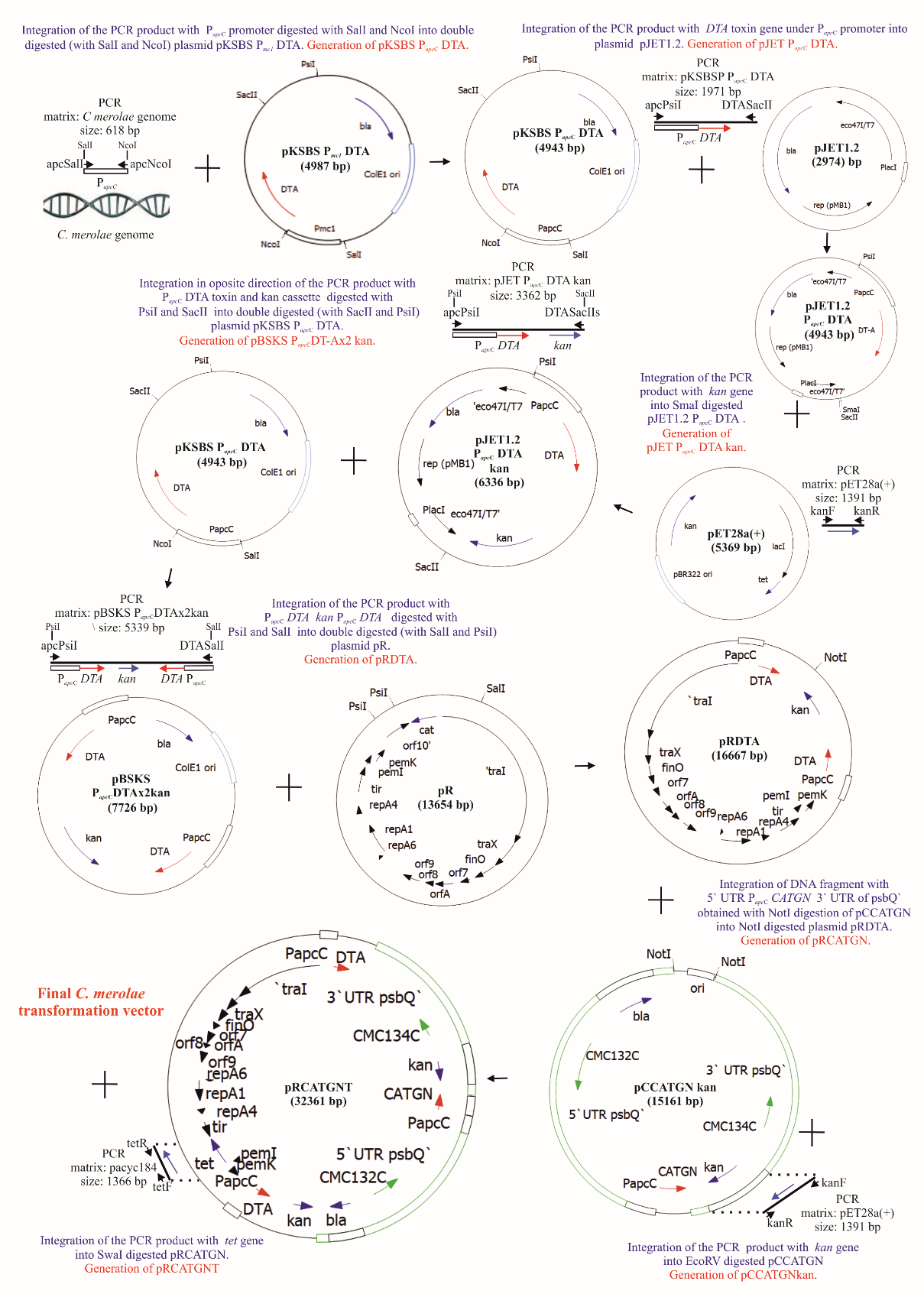


**Figure S1.** **The construction scheme of the pRCATGNT transformation vector**. In navy blue –resistance cassettes (*kan,tet,bla,cat* - resistance to kanamycin, tetracycline, ampicillin, chloramphenicol, respectively). In red - codon optimized selection marker catgn gene (resistance to chloramphenicol) under promoter of the *apc*C gene (CMO250C, phycocyanin-associated rod linker protein) g (P*_apc_*_C_) and *Diphtheria* toxin A genes (DTA). In green – C. merolae sequence with two identified genes: CMC132C (*rpl*O, mitochondrial ribosomal protein L15 precursor), CMC134C (probable constituent of 66S pre-ribosomal particles Nsa2p). In black – plasmid vector genes. Plasmid diagrams not in scale.
