# Supplementary material for: Deletion of psbQ’ gene in Cyanidioschyzon merolae reveals the function of extrinsic PsbQ’ in PSII
Source: Plant Mol Biol. 2017 Dec 1;96(1):135–49. doi: 10.1007/s11103-017-0685-6 (PMC5778172; doi:10.1007/s11103-017-0685-6)
Supplement: Supplementary file 5 — Supplementary material 5 (DOCX 109 KB) [file 11103_2017_685_MOESM5_ESM.docx]

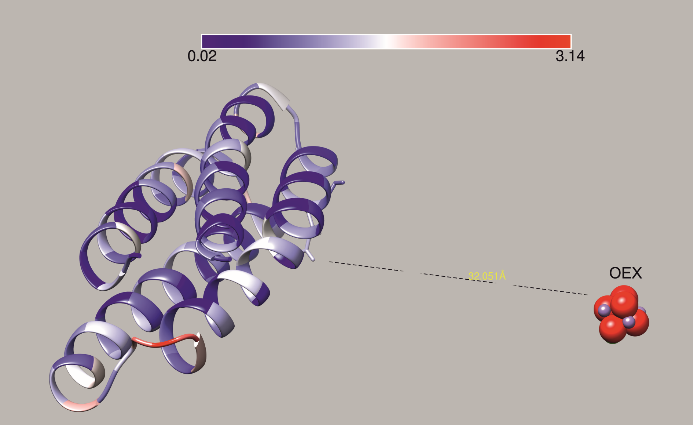


**Figure S2.** **Modeled structure of *C. merolae* PsbQ’.**

The protein structure was generated by Chimera 1.11.2 software and the “Modeler” subroutine. The recently solved structure of *C. caldarium* PSII (Protein Data Bank coordinates 4YUU; Ago et al. 2016) was used as the scaffold for modeling. The 4YUU PsbQ’ structure provides coordinates only for amino acids in the range between 103-160 and 165-217 out of 218. Analogously, the *C. merolae* modeled coordinates ranged between 102-159 and 164-216 out of 217. The average distances between residues of the modeled and the scaffold structure ranged between 0.02 and 1.49 Å (rendered in blue-white tones) with the noticeable exception of the 160-163 residues at the vertex between the II and the III helix (rendered in red-white tones). At this point, the 4YUU structure is discontinuous and distanced between residues are based on modeled scaffold structure The distance from the OEC (OEX molecule) to the nearest residue of the PsbQ’ protein is 32.05 Å, practically excluding any direct interactions of PsbQ’ with OEC.
