# Supplementary material for: Deletion of psbQ’ gene in Cyanidioschyzon merolae reveals the function of extrinsic PsbQ’ in PSII
Source: Plant Mol Biol. 2017 Dec 1;96(1):135–49. doi: 10.1007/s11103-017-0685-6 (PMC5778172; doi:10.1007/s11103-017-0685-6)
Supplement: Supplementary file 6 — Supplementary material 6 (DOCX 1052 KB) [file 11103_2017_685_MOESM6_ESM.docx]

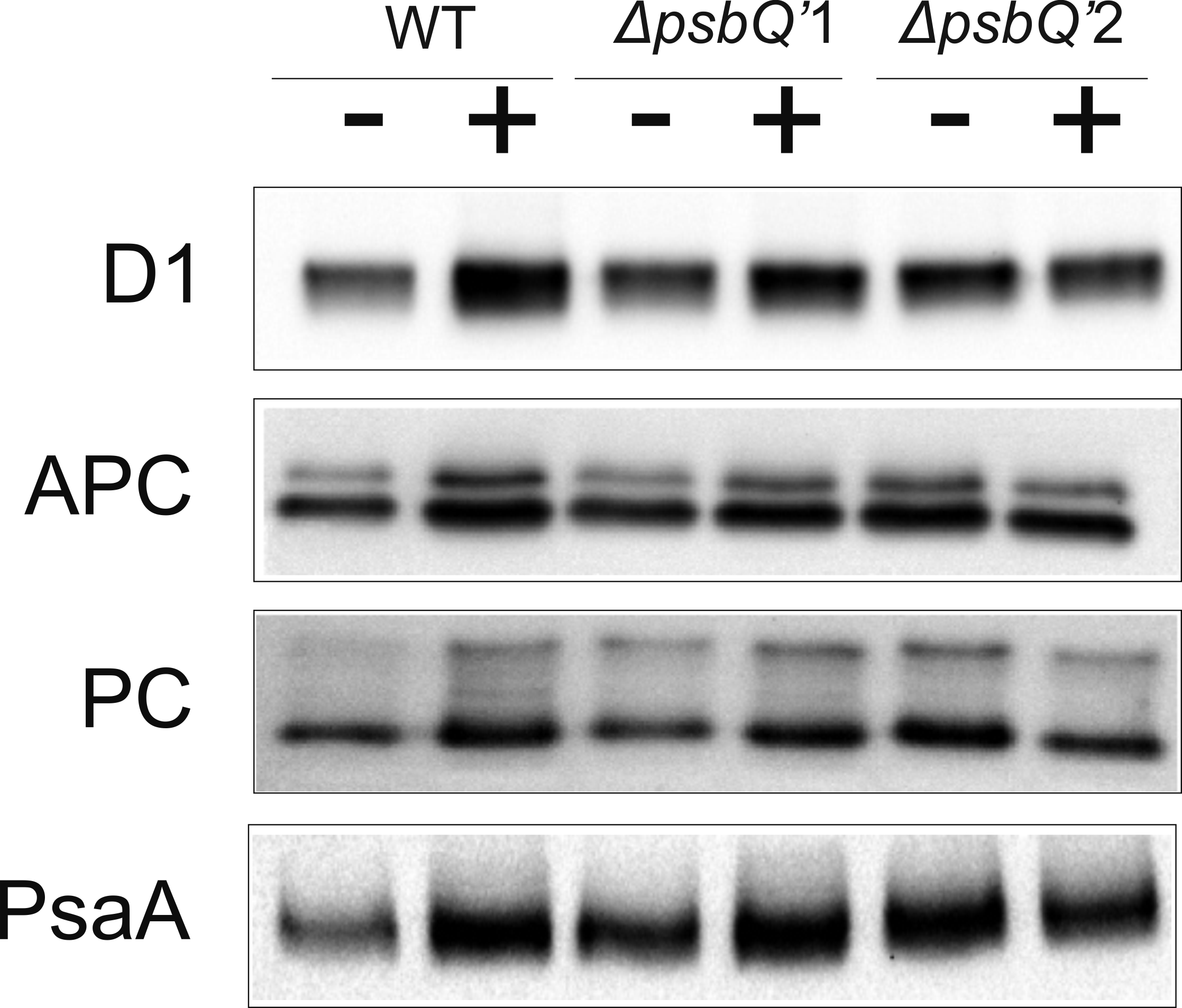


**Figure S3.** **Western blot of WT and both mutant cells.**

Equal number of mutant and WT cells was taken for the SDS-PAGE, followed by Western blot hybridization with anti-D1, anti-APC, anti-PC and anti-PsaA antibody. Cells were grown in atmospheric CO_2_ concentration of 0.05% (-) and in 5% (+) CO_2_ chamber.
